# Identification of *Stk25* as a Genetic Modifier of Tau Phosphorylation in *Dab1*-Mutant Mice

**DOI:** 10.1371/journal.pone.0031152

**Published:** 2012-02-15

**Authors:** Tohru Matsuki, Mariam Zaka, Rita Guerreiro, Marcel P. van der Brug, Jonathan A. Cooper, Mark R. Cookson, John A. Hardy, Brian W. Howell

**Affiliations:** 1 Neuroscience and Physiology, State University of New York Upstate Medical University, Syracuse, New York, United States of America; 2 Neurogenetics Branch, National Institute of Neurological Disorders and Stroke, National Institutes of Health, Bethesda, Maryland, United States of America; 3 Laboratory of Neurogenetics, National Institute on Aging, National Institutes of Health, Bethesda, Maryland, United States of America; 4 Division of Basic Sciences, Fred Hutchinson Cancer Research Center, Seattle, Washington, United States of America; National Institute of Health, United States of America

## Abstract

Hyperphosphorylation of the microtubule binding protein Tau is a feature of a number of neurodegenerative diseases, including Alzheimer's disease. Tau is hyperphosphorylated in the hippocampus of *dab1*-null mice in a strain-dependent manner; however, it has not been clear if the Tau phosphorylation phenotype is a secondary effect of the morbidity of these mutants. The *dab1* gene encodes a docking protein that is required for normal brain lamination and dendritogenesis as part of the Reelin signaling pathway. We show that *dab1* gene inactivation after brain development leads to Tau hyperphosphorylation in anatomically normal mice. Genomic regions that regulate the phospho Tau phenotype in *dab1* mutants have previously been identified. Using a microarray gene expression comparison between *dab1*-mutants from the high-phospho Tau expressing and low-phospho Tau expressing strains, we identified Stk25 as a differentially expressed modifier of *dab1*-mutant phenotypes. Stk25 knockdown reduces Tau phosphorylation in embryonic neurons. Furthermore, Stk25 regulates neuronal polarization and Golgi morphology in an antagonistic manner to Dab1. This work provides insights into the complex regulation of neuronal behavior during brain development and provides insights into the molecular cascades that regulate Tau phosphorylation.

## Introduction

The Reelin signaling pathway regulates neuronal placement and dendritogenesis during brain development and long-term potentiation (LTP) in adults [1]–[4]. Reelin is a glycoprotein that is expressed in the neocortex, hippocampus, cerebellum and spinal cord [5], [6]. It signals through two partially redundant LDL-super family receptors, VLDLR and APOER2 [7]–[9]. Reelin-regulated receptor dimerization recruits an essential docking protein, Dab1, to the cytoplasmic tails of these receptors, activates Src family kinases and induces Dab1 tyrosine phosphorylation [10]. As a consequence, a number of downstream signaling axes that are required for normal brain development are activated [11]–[15].

Interestingly, there are numerous connections between the components of the Reelin signaling pathway and molecules that have been implicated in Alzheimer's disease (AD). The Reelin receptors are ApoE receptors, and the ApoE4 allele is a risk factor for late onset AD [16]. Dab1 binds to the C-terminus of the amyloid precursor protein (APP) [17], [18], the precursor to the pathogenic Aβ peptide, and influences APP cleavage [19] Furthermore, Dab1 acts downstream of APP on a developmental pathway [20]–[22]. Expression of Reelin and Dab1 are increased in the forebrains of AD patients [23], [24]. However Reelin expression is decreased in the entorhinal cortex of these patients [25]. Reelin binds to the extracellular domain of APP and regulates its cleavage [20]. Reelin also relieves the inhibition of synaptic transmission caused by Aβ treatment [26]. Reducing the Reelin gene dose augments Aβ plaque formation in a mouse model of Alzheimer's disease [27]. In addition, homozygous inactivation of the genes encoding the Reelin signaling pathway leads to Tau hyperphosphorylation, which is a hallmark of AD [8], [28].

Tau is a microtubule binding protein that increases microtubule stability and hinders the movement of plus- and minus-end directed microtubule- dependent motor proteins [29], [30]. Its phosphorylation releases Tau from microtubules, allowing unhindered movement of the motor proteins. Tau is highly phosphorylated during development, reflecting the dynamic cytoskeletal changes that are occurring at this time. Excessive Tau phosphorylation, however, promotes the formation of neurofibrillary tangles, which are evident in diseased neurons in the brains of Alzheimer's patients at autopsy [31], [32]. Tau is believed to play a role in the pathology of several other neurodegenerative disorders including Parkinson's and Frontal Temporal Lobe Dementia [33]. A number of kinases are known to regulate Tau phosphorylation; however, their regulation in diseased brain is not fully understood.

Here we show that inactivating the *dab1* gene in postnatal animals leads to Tau hyperphosphorylation in the hippocampus, and we identify a genetic modifier of *dab1* that regulates this process. A previous genome-wide screen identified three quantitative trait loci (QTLs) that cosegregated with Tau phosphorylation in homozygous *dab1* (−/−) mutants [28]. We investigated whether modifier genes within these QTLs could be identified by differences in mRNA levels between high-phospho Tau expressing C57BL/6 *dab1* versus the low-phospho Tau expressing BALB/c *dab1* mutant mice. We identified Stk25, an Ste20-like serine/threonine kinase, as a differentially expressed gene that maps to one of the QTLs. Knocking-down Stk25 expression reduces Tau phosphorylation, and as we have recently shown, Stk25 regulates neuronal polarization and Golgi morphology in a competitive manner with Reelin-Dab1 signaling [34].

## Results

### *Dab1* gene inactivation in postnatal animals leads to Tau hyperphosphorylation in the hippocampus

Tau hyperphosphorylation in the hippocampus is a strain-dependent *dab1* mutant phenotype [28]. Increased Tau phosphorylation is observed in C57BL/6 or mixed C57BL/6-129SV *dab1* homozygous (*dab1* −/−) mutant mice at postnatal day 19 (P19). These animals die shortly after weaning. In contrast, BALB/c-background *dab1* mutants have little or no detectable Tau phosphorylation and have more normal lifespans. It is therefore not clear whether the augmented Tau phosphorylation is a result of inactivating the Reelin-Dab1 pathway, or if it is secondary to the morbidity of the *dab1*-mutant mice on the backgrounds with observed Tau phosphorylation.

To resolve this issue, we inactivated the *dab1* gene in a mutant mouse line with a conditional *dab1* (*dab1 cKI*) gene. These animals have been described previously and have relatively normal brains when *dab1* is inactivated after birth [14], [21]. The *dab1* gene was inactivated by tamoxifen injection in animals homozygous for the *dab1 cKI* (dab1^cKI/cKI^) allele and carrying a ubiquitously expressed, tamoxifen-inducible Cre transgene (Cre^ERTM^) [35]. We did not observe any aberrant behavior or increased mortality in animals with a conditionally inactivated *dab1* gene as compared to controls. Tau phosphorylation in this treatment group was compared to tamoxifen-treated *dab1 cKI* homozygous animals that lack the Cre transgene to control for any non-specific effects of tamoxifen.

**Figure 1:**
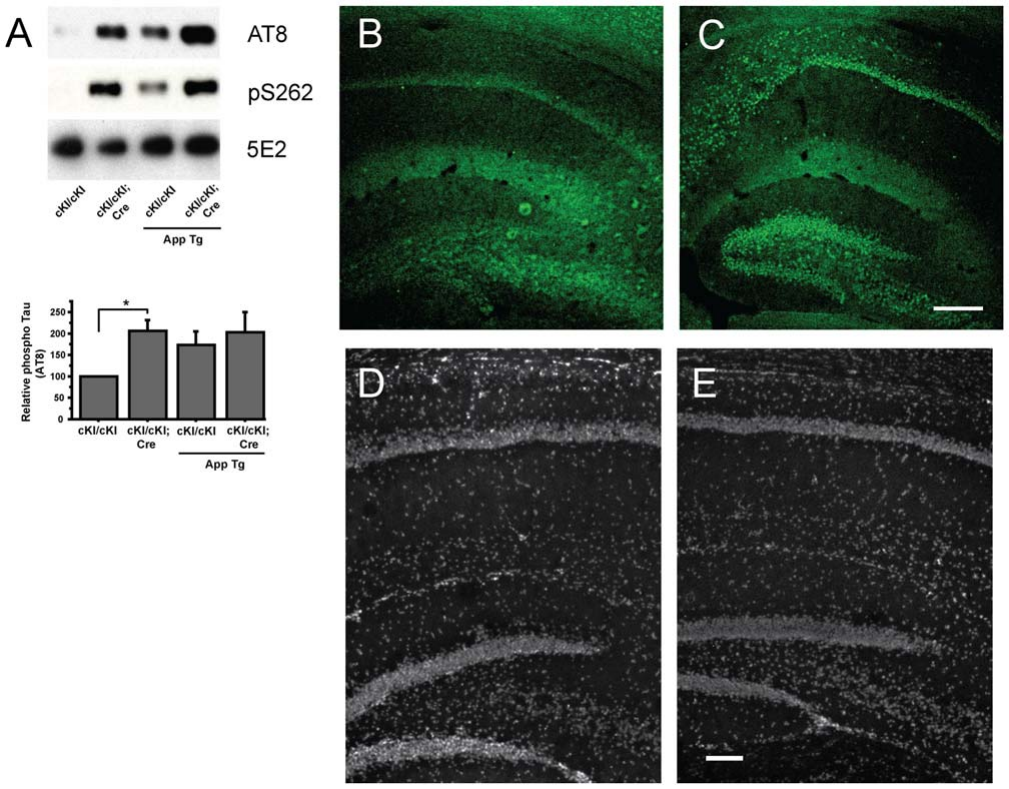
Postnatal inactivation of the *dab1* gene leads to Tau hyperphosphorylation in hippocampal neurons. **A** Phospho Tau levels at Ser202/Thr205 (AT8 site) and Ser 262 were increased in *dab1^cKI/cKI^*; *ESR-Cre^ERTM^* animals as compared to control *dab1^cKI/cKI^* animals that were treated with tamoxifen at P11 and sacrificed on P40. The presence of an APP^SWE^ transgene did not increase the phospho Tau levels in *dab1*-deficient mice. Total Tau levels were relatively constant (5E2 immunoblot). Tau phosphorylation levels (AT8 site) normalized to total Tau were compared between hippocampal lysates of 3 sets of animals (* p = 0.01, Student's t-test). **B** Background levels of phospho Tau were observed by AT8 antibody immunohistochemical analysis of tamoxifen-treated (P7) *dab1^cKI/cKI^* hippocampi at P40. **C** Tau phosphorylation was observed in the soma of hippocampal neurons of *dab1^cKI/cKI^*; *ESR-Cre* mice treated in the same manner. **D**, **E** DAPI stained sections of tamoxifen-treated (P7) *dab1^cKI/cKI^* and *dab1^cKI/cKI^*; *ESR-Cre* hippocampi, respectively. Error bars indicate standard error of the mean (SEM) in all figures. Bar = 200 µm C, 100 µm E.

Dab1 expression was surveyed between the experimental and control mice in hippocampal cell lysates. Cre^ERTM^ activation by tamoxifen is known to be only partially penetrant and in most hippocampi, Dab1 expression was reduced to approximately 50% by Cre-lox recombination (data not shown). Brains that did not show at least 40% reduction in Dab1 expression were excluded from further analysis. Tau phosphorylation was significantly increased at P40 by *dab1* gene inactivation at P11 in the brains of Cre^ERTM^ transgenic *dab1^cKI/cKI^* animals (Fig. 1A) as compared to the tamoxifen-treated control animals. Increases in phosphorylation were observed at both the AT8 (Ser202/Thr205) and Ser262 sites. Interestingly, the augmented Tau phosphorylation was observed to localize to the cell soma of neurons in CA1–CA3 and in the dentate gyrus (compare Fig. 1C to 1B). The hippocampal histology of tamoxifen-treated *dab1^cKI/cKI^* and *dab1^cKI/cKI^*; *Cre^ERTM^* mice appeared to be relatively normal (Fig. 1D, 1E). Tau phosphorylation is qualitatively different from that observed in *dab1*-null animals where Tau hyperphosphorylation is localized to the nerve tracts, particularly in the dentate gyrus [28].

Overexpression of the Swedish mutant amyloid precursor protein (APP^SWE^) has been shown to augment Tau phosphorylation in lines of mice that are sensitized by expression of additional proteins such as Tau and Psen1 [36]. We therefore tested to see if APP^SWE^ expression augments the Tau phosphorylation phenotype observed by *dab1* gene inactivation. On average APP^SWE^ overexpression had no effect on Tau phosphorylation in mice with inactivated *dab1* genes (Fig. 1A). There was an increase in phospho Tau levels in *dab1* wild-type mice overexpressing APP^SWE^; however, this was not statistically significant.

Thus Tau hyperphosphorylation in hippocampal neurons of *dab1* mutant mice appears to be a direct or indirect consequence of loss-of-*dab1* function and not a secondary effect related to the weakened condition of *dab1*-null mice. Modifier genes are thought to regulate the strain dependence of the Tau phosphorylation phenotype [28].

### Identification of a modifier of the *dab1*−/− Tau hyperphosphorylation phenotype

To identify genes that regulate the Tau phosphorylation phenotype in *dab1−/−* mutants, we selected genes that are differentially expressed between *dab1−/−* mutants on the C57BL/6 and BALB/c strain backgrounds that map in the vicinity of previously identified QTLs [28] (Table 1). Using microarray analysis, gene expression was examined in the hippocampus at P19, an age when Tau hyperphosphorylation is observed in C57BL/6 strain, *dab1*-mutant mice. From seven differentially expressed genes that map near to the QTL on chromosome 1, we selected *stk25* for further study. It encodes an Ste20-like serine/threonine kinase and maps within 2 Mb of the D1Mit365 polymorphism (Table 2). Clear candidate modifiers were not identified near the other QTLs. By microarray analysis, Stk25 was expressed 1.9 fold higher in *dab1* mutants on the C57BL/6 versus the BALB/c background. A similar fold difference in expression was observed between wild-type animals of the same strains.

**Table 1:** Strain-dependent expression of genes in the mouse hippocampus.

	genomic locus	differentially expressed gene
wild type: C57BL/6J vs BALB/c	all	588
	all	961
*dab1 KO*: C57BL/6J vs BALB/c	Chromosome 1 (D1Mit215-D1Mit90)	7
	Chromosome 12 (D12Mit12-D12Mit18)	40
	Chromosome 16 (D16Mit94-D16Mit86)	0

We examined expression of mRNA transcripts by microarray between C57BL6 and BALB/c wild-type or *dab1−/−* mutant mice. Genes that were differentially expressed above a threshold of 1.5 fold were scored and examined for chromosomal position related to QTLs that are associated with Tau hyperphosphorylation in *dab1*−/− mutants (Brich et al., 2003).

**Table 2:** Characterization of genes that are differentially expressed between C57BL6 and BALB/c background Dab1 mutant mice and map to the Tau phosphorylation QTL on chromosome 1.

Gene Symbol	Accession number	Distance from D1Mit365 (Mb)	fold differences	Gene description
Scly	BC019879	0.83	−3.76	Putative selenocysteine lyase
Stk25	BC071218	1.49	1.86	Serine/threonine-protein kinase 25
Thap4	BC013538	1.58	2.29	THAP domain containing 4
Vps4b	BC003799	14.63	3.17	vacuolar protein sorting 4B
Clasp1	BC057312	26.26	2.39	cytoplasmic linker associated protein 1
Dbi	BC028874	27.98	−1.68	diazepam binding inhibitor (GABA receptor modulator, acyl-Coenzyme A binding protein)
Dpp10	BC067026	31.20	1.65	dipeptidyl-peptidase 10

The QTL at D1Mit365 segregates with Tau hyperphosphorylation in *dab1*−/− mutant mice. We identified 7 differentially expressed genes that map between markers that define the outer limits of the QTL (D1Mit215-D1Mit90). One of these, Stk25, was considered a good candidate since it encoded a serine/threonine kinase. Gene expression differences for *dab1−/−* mutant animals are shown for C57BL/6 versus BALB/c backgrounds (negative value indicates higher expression in BALB/c).

**Figure 2:**
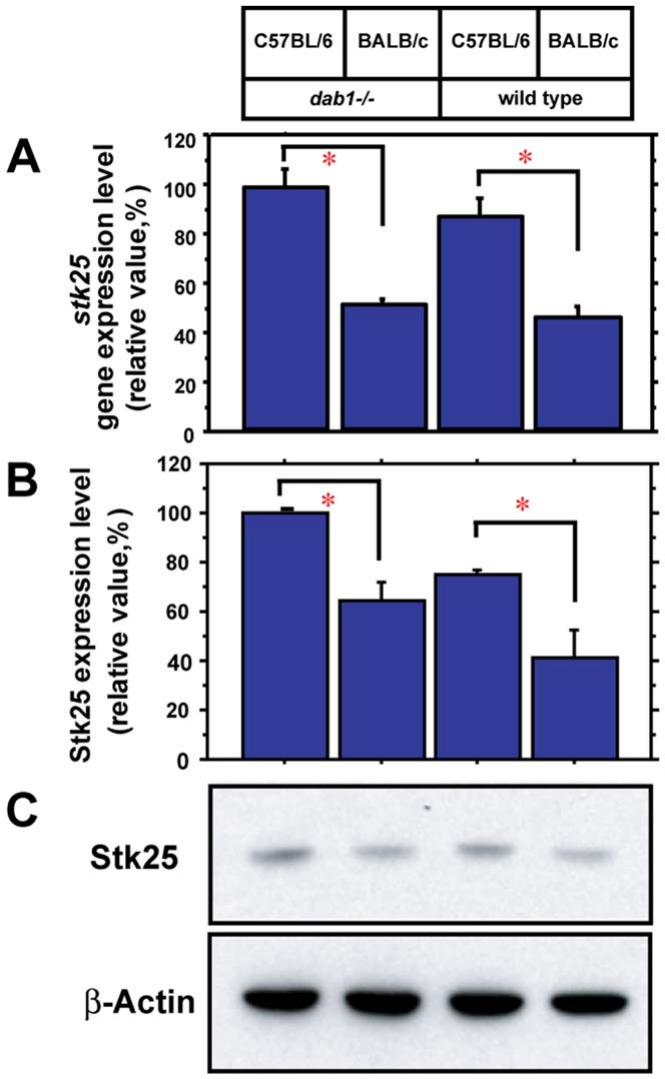
Stk25 mRNA and protein levels are strain-dependent. **A** Stk25 mRNA expression was 1.8-fold higher in C57BL/6 versus BALB/c strain in *dab1* −/− and wild-type mice by real-time PCR. **B** Western blot analysis of Stk25 protein levels showed higher levels of expression in *dab1* −/− and wild-type mutant animals on the C57BL/6 than the BALB/c strain. **C** Representative anti-Stk25 (upper panel) and anti-actin (lower panel) Western blots of hippocampal lysates.

To verify whether Stk25 expression is dependent on the mouse strain, we characterized it by quantitative real-time polymerase chain reaction (RT-PCR) and immunoblotting (Fig. 2). Consistent with the microarray data, Stk25 was approximately 1.8 fold more highly expressed in C57BL/6 versus BALB/c mice, independent of the *dab1* genotype (Fig. 2A). By Western blotting, we found that Stk25 protein expression was about 1.6 fold higher in C57BL/6 than BALB/c *dab1−/−* mice (Fig. 2B, 2C). We observed greater Stk25 protein expression in *dab1* −/− mutants versus wild-type animals (p<0.05), which could reflect a change in Stk25 stability or translation in the absence of Dab1. Therefore, Stk25 mRNA and protein expression is significantly higher in *dab1* mutants from the C57BL/6 strain that display Tau hyperphosphorylation than the BALB/c strain that does not.

**Figure 3:**
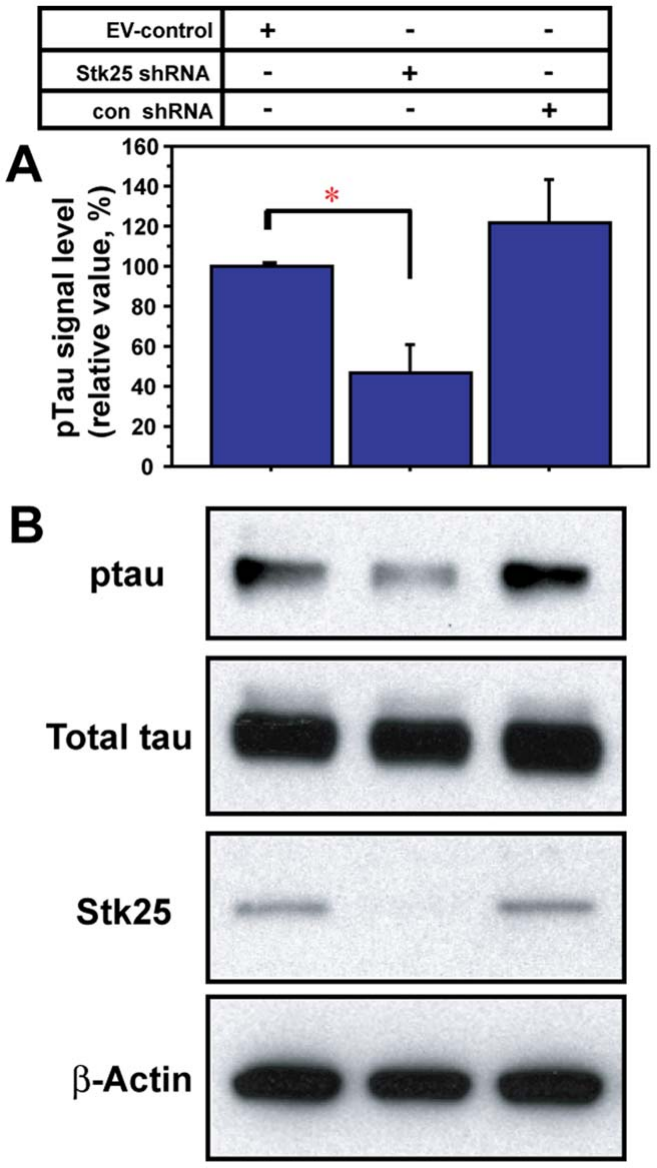
Stk25 knockdown reduces Tau phosphorylation in embryonic neurons. **A** Phospho-Tau normalized to total Tau was significantly reduced in primary neurons infected with Stk25 shRNA viruses compared to neurons infected with either an empty vector (EV)-control or the control shRNA (con shRNA). **B** Tau phosphorylation at pSer202/pThr205 (AT8 antibody, 1^st^ panel) was lower in samples with reduced Stk25 expression (3^rd^ panel), whereas total Tau levels (2^nd^ panel) and β-actin levels (4^th^ panel) were unchanged. (* p<0.05, Student's t test, n = 3).

Since Stk25 is not known to regulate Tau phosphorylation, we examined the effect of reducing Stk25 in embryonic neurons, where Tau proteins are highly phosphorylated (Fig. 3). We engineered a lentivirus to express a GFP marker and an Stk25 shRNA that reduces Stk25 expression by approximately 80% (Fig. 3B) [34]. The control shRNA has three mismatches in the stem-loop structure and did not reduce Stk25 expression, nor did the empty vector (EV)-control. In the Stk25 knockdown samples, significantly lower AT8 site (pSer202/pThr205) Tau phosphorylation was observed as compared to the EV-control and control Stk25 shRNA-infected neuronal cultures, while total Tau levels were not affected (Fig. 3A, 3B). These results suggest that Stk25 expression correlates with augmented Tau phosphorylation in postnatal *dab1−/−* mutant brain and embryonic neurons in culture.

**Figure 4:**
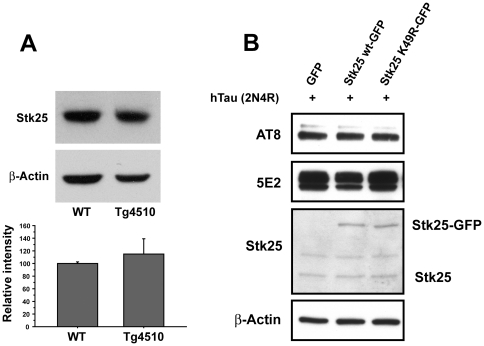
Stk25 expression is not correlated with augmented Tau phosphorylation in a Tauopathy model or in transfected HeLa cells. **A** Lysates from brains of 9.5 month old wild-type or Tg4510 mutant animals were immunostained for Stk25 and β-actin (upper panel). The average Stk25 signal normalized to β-actin from 3 separate samples from each genotype showed no statistically significant difference (lower panel). **B** Overexpression of Stk25-GFP did not lead to increased phosphorylation of co-transfected Tau in HeLa cells. Antibodies used for Westerns indicated at left: AT8 (phospho Ser202/Thr205 Tau) 5E2 total Tau. Sizes of expected Stk25-GFP and endogenous Stk25 are indicated at right.

To determine if Stk25 expression correlates with augmented Tau phosphorylation in disease conditions, we examined its expression in a tauopathy model mouse strain (Tg4510) that overexpresses human mutant Tau (P301L) [37], [38]. Tau expression in these mice is dependent upon an activator gene that is suppressed by doxycycline. In this study, mice that have the activator transgene (control) and those that have both the activator and human Tau P301L genes were compared for Stk25 expression. Transgenic mice that express both the activator and Tau P301L transgenes show increased levels of Tau phosphorylation at the AT8 site and develop neurofibrillary tangles by 7 months of age [37]. Stk25 expression was, however, unchanged between control and affected Tg4510 mice at 9.5 months (Fig. 4). This demonstrates at least in this model that Stk25 does not play a role to augment Tau phosphorylation.

We then assessed whether Stk25 overexpression would augment Tau phosphorylation. HeLa cells were cotransfected with Stk25-GFP and human Tau. This led to an approximate twofold increase in Stk25 expression (Fig. 4B). Two days after transfection the cells were lysed, proteins were resolved by SDS-polyacrylamide gel electrophoresis and immunobotted with antibodies against phospho-Tau (AT8), total Tau, Stk25 and β-actin. We did not observe any increased Tau phosphorylation in samples that overexpressed wild-type Stk25 as compared to those that overexpressed kinase-inactive Stk25 or expressed only endogenous levels of Stk25. This suggests that Stk25 overexpression alone is not sufficient to augment Tau phosphorylation.

**Figure 5:**
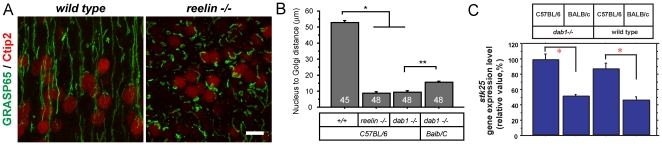
The Golgi extension and neuronal polarization phenotypes in *dab1* mutant neurons is dependent upon mouse strain background. The appearance of the Golgi apparatus was examined in 100 µm thick sections of the hippocampus at birth by immunostaining for GRASP65 and Ctip2 to identify pyramidal neurons. **A** In wild-type animals the Golgi apparatus extends several microns into the presumptive apical dendrite in the hippocampi, but in C57BL/6 *dab1−/−* mutants it is convoluted near the nucleus. The Golgi appears more elongated in the Balb/c *dab1−/−* mutant mice. Nucleus to Golgi distances were measured on isolated cells (insets). Arrowheads represent points used for measurements. **B** The nucleus to Golgi tip distances are greater for wild-type than *dab1−/−* mutants, and greater for BALB/c versus C57BL/6 *dab1−/−* mutants (*,** p<0.0001, Student's t test). **C** The number of multiple axon bearing neurons in *dab1−/−* mutant neurons is reduced on the BALB/c background as compared to the C57BL/6 background. In both cases, knocking down Stk25 leads to a further reduction in neurons with multiple axons and the development of neurons with no axons (* p<0.001 compared to the respective EV-control samples, ** p = 0.01 compared between EV-control samples, Student's t test) (Bar = 20 µm) Values for C57BL/6 samples have been published previously [34] and are shown here for comparison to BALB/c samples.

We have recently shown that Reelin-Dab1 signaling and Stk25 have antagonistic roles to regulate Golgi deployment and neuronal polarization [34]. Since BALB/c mice express less Stk25 than C57BL/6 mice, it is possible that Golgi extension and neuronal polarization phenotypes differ between neurons on these two backgrounds. We examined the appearance of the Golgi apparatus in the Ctip2-positive hippocampal pyramidal neurons in *dab1*−/− mutants to determine if this phenotype is dependent upon the background. As shown previously, the Golgi apparatus in C57BL/6 *dab1*−/− mutant hippocampi is considerably more convoluted than the wild-type control (Fig. 5A, 5B) [34]. Interestingly, the Golgi is significantly more extended in BALB/c *dab1−/−* mutant pyramidal neurons than in corresponding neurons from C57BL/6 strain mutants (Fig. 5A, 5B). Golgi extension in hippocampi of wild-type animals was similar between the BALB/c and C57BL/6 strains (data not shown).

Neurons from C57BL/6 *dab1*−/− mice have a greater propensity to extend multiple axons than wild-type neurons. Axon production was reduced by knocking down Stk25 levels [34]. Approximately 37%+/−3% of *dab1* mutant neurons from C57BL/6 mice extend multiple neurons as compared to 19%+/−3% of BALB/c *dab1−/−* mutant neurons (Fig. 5C). The number of multiple axon bearing neurons was also further reduced by Stk25 shRNA expression in BALB/c strain mice. This treatment also increased the number of neurons with no axon from 0% to 38%+/−6% and 39%+/−7% for C57BL6 and BALB/c neurons, respectively. Together this suggests that differential expression of Stk25 or another modifier gene between the two strains is capable of modulating the Golgi extension and neuronal polarization phenotypes.

Obvious candidate modifiers were not identified within the QTLs on chromosomes 12 and 16. This could suggest that putative modifiers within these loci are not differentially expressed. Previously we highlighted APP and Psen1 as good candidate modifiers in these regions since they have known links to Alzheimer's disease and Tau hyperphosphorylation [28], however, we did not detect significant differences in expression of APP or Psen1 between C57BL/6 and BALB/c samples.

One possibility is that other modifiers are regulated by sequence differences. We therefore determined the exon sequence of APP in C57BL/6 and BALB/c mice.

A deletion of 9 bp (3xGGC) in exon 1 of *App*, located 73 bp before the beginning of the initiator methionine was found in C57BL/6 mouse strain when compared to the sequence of BALB/c *App* (Table 3). This variant does not create a new start codon. A difference in exon 5 was also found, giving rise to a synonymous change (H166H G<>A). These variants have not previously been reported in mice or humans.

**Table 3:** Sequence comparison of exon I of the *APP* gene from C57BL/6 and BALB/c mice.

BALB/c	A C C G G A G A C **G G C G G C G G C G G C G G C G G C G G C** G C G G
C57BL/6	C G C G G G G C C A C C G G A G A C **G G C G G C G G C G G C** G C G G

The GCG repeated sequence (bold) begins 73 bp upstream of the initiator Met. There are 7 repeats in this region of the *APP* gene in BALB/c mice as compared to 4 in C57BL/6.

## Discussion

We show here that *dab1* gene inactivation in healthy animals with relatively normal brain anatomy leads to Tau hyperphosphorylation. In addition, we identify Stk25 as a differentially expressed gene located within a previously identified QTL that cosegregates with Tau hyperphosphorylation in *dab1*-null mice. Higher Stk25 expression in C57BL/6 *dab1* mutant mice correlates with the high Tau phosphorylation observed in these mice as compared to BALB/c *dab1*-mutant mice. Knocking down Stk25 reduces Tau phosphorylation in embryonic neurons in culture (Fig. 3). Overexpressing Stk25 with human Tau in HeLa cells, however, does not increase Tau phosphorylation (Fig. 4B). Furthermore Stk25 expression was not augmented in a tauopathy model mouse line (Fig. 4A). Since we evaluated this by Western blotting, it remains possible that Stk25 is upregulated in a subset of neurons in these mice. However, it seems likely that Stk25 does not directly phosphorylate Tau and that unknown cofactors are required to link Stk25 signaling to Tau phosphorylation. Alternatively the high tau phosphorylation observed in *dab1* mutant mice may be an indirect consequence of high Stk25 expression. For instance it could be the result of neuronal compensation to the cellular perturbation caused by loss of Reelin-Dab1 signaling in the presence of high Stk25 levels. Lower Stk25 expression in BALB/c neurons is correlated with less severe Golgi extension and neuronal polarization phenotypes in *dab1*-mutants from this strain as compared to mutants on the C57BL/6 background (Fig. 5). Knocking down Stk25 levels in *dab1*-mutant neurons in both backgrounds leads to a restoration of normal neuronal polarization. This suggests that Stk25 levels influence *dab1*-mutant phenotypes, including Tau phosphorylation and Golgi extension.

Stk25 is an Ste20 family kinase that we have recently shown acts downstream of LKB1-STRAD and upstream of GM130 to regulate cell polarization and Golgi morphology [34]. The LKB1 pathway is known to regulate Tau phosphorylation through the MARK and SAD/BRSK kinases [39]–[42]. Interestingly, the kinase activity of Stk25 is not required for its role in cell polarization or Golgi regulation [34]. It is possible that in this capacity it acts as a scaffolding protein to link LKB1 signaling to the Golgi apparatus where it may regulate GM130 function. Stk25 overexpression reduces the extent of Reelin-induced Golgi deployment. Congruent with this, we found that *dab1*−/− BALB/c strain mice, which express less Stk25, have significantly more extended hippocampal Golgi apparatuses than *dab1*−/− C57BL/6 strain mice. A similar strain dependency was observed for the multiple axon phenotype of *dab1* mutants. In C57BL/6 mice, approximately 30 percent of *dab1* −/− hippocampal neurons had multiple axons as compared to only 20 percent in primary cultures of BALB/c neurons. This suggests that the Reelin-Dab1 pathway and Stk25 and potentially other modifiers either converge on a common downstream molecular target or regulate distinct biological phenomenon that have the opposite influences on Golgi morphology and neuronal polarization.

Testing for expression differences between strains did not lead us to the identification of modifier genes in the QTLs on chromosomes 12 and 16. Within the chromosome 12 QTL there were 40 differentially expressed genes, too many to easily test for regulation of Tau phosphorylation. These await further analysis. In contrast there were no differentially expressed genes within the chromosome 16 QTL. The exon sequence of APP, a candidate modifier within the chromosome 16 QTL, revealed differences between the C57BL/6 and BALB/c strains. The chromosome 16 QTL from BALB/c mice correlated with higher Tau phosphorylation in mice [28]. In the BALB/c mouse, exon 1 has a total of 7 GGC repeats, three more than are present in the C57BL/6 mice. The human sequence is distinct from either mouse strain with 2 pairs of repeats interrupted by GGT (2XGGC-GGT-2XGGC). It is possible that this region in the 5′ UTR may regulate translation of the APP mRNA. Interestingly, in the *reelin* gene there is also a polymorphic GGC repeat in the 5′ UTR and the longer repeat has been shown to downregulate translation of a luciferase reporter gene [43]. Based on this we would anticipate that APP translation could be higher in C57BL/6 than BALB/c mice. Higher levels of *APP* gene dose and presumably APP expression are correlated with the increased likelihood of developing AD [44]. It remains to be determined, however, whether the configuration of GGC repeats in the APP gene regulates translation in the brain.

In summary, *dab1* mutant mice provide an interesting model of human Tauopathies. In these mice, overexpression of Tau and other disease genes is not necessary to observe increased Tau phosphorylation, which we show here is caused by *dab1* gene inactivation in the absence of severe developmental disease. Several studies have implicated Reelin-Dab1 signaling with molecules associated with AD. Paradoxically Reelin and Dab1 levels have been shown to be elevated in the forebrain of AD patients, while a loss-of-function *Reelin* mutation appears to accelerate the disease progression in a mouse model. Thus more work is required to elucidate the role of this pathway in AD [23], [24], [27]. Identifying Stk25 as a modifier of this pathway, which regulates Golgi morphology and neuronal polarization, provides a new perspective to evaluating the cause and consequences of Tau hyperphosphorylation. Since Stk25 has an opposing role to Reelin-Dab1 signaling, it will be interesting to determine if Stk25 influences the progression of neurodegeneration in mouse models of AD.

## Materials and Methods

### Animals

The *dab1* conditional mice (*dab1^cKI^*) were maintained on a mixed C57BL/6/SV129 background and have been described in detail previously [21]. Briefly, a floxed cDNA cassette encoding residues 23–555 was knocked into the *dab1* locus to replace exon III of the *dab1* gene. The knock-in event also introduced a beta-galactosidase reporter and a PGK-neomycin cassette that is flanked by frt sites. The neomycin cassette was removed in the mice used in this manuscript by breeding the animals with a flp-deleter strain. Loss of PGK-neo was confirmed by PCR. The animals were then back crossed to C57BL/6 and selected against the flp transgene. Trangenic mice that ubiquitously express the tamoxifen inducible Cre fusion (Cre^ERTM^) have been described previously and were obtained from Jackson Laboratories [35]. Tamoxifen (Sigma) was dissolved in corn oil (20 mg/mL) and intraperitoneal injections (225 µg/g animal weight) were given at P11 and animals were perfused with 4% paraformaldehyde in PBS at P40. The *dab1*-null allele has been described previously and was maintained as congenic C57BL/6 and BALB/c strains [45]. The APP^SWE^ transgenic animals (TG2576; B6/SJL background) [46] were obtained from Taconic Laboratories and crossed with the *dab1^cKI^* mice to generate animals of the indicated genotypes.

### Ethics Statement

All animals in this study were treated humanely according to animal care and use guidelines of the NIH. The use of animals for this study was approved by the National Institute of Neurological Disorders and Stroke-Institutional Animal Care and Use Committee (A4149-01; approval number 1078-05) and the State University of New York Upstate Medical University-Committee for the Humane Use of Animals (A3514-01; approval number 108).

### Antibodies and Vectors

The following anti-Tau antibodies were employed where indicated: 5E2 (total Tau; Upstate Biotechnology, Lake Placid, NY), AT-8 (phosphoSer 202/Thr205; Pierce Biotechnology, Rockford, IL) and Tau phosphoSer262 (Sigma). Stk25 was detected with anti-YSK antibody (Santa Cruz), and actin was detected with anti-actin (20–33) (Sigma). Axons were identified with pan-axonal neurofilament marker SMI-312 (Covance), the Golgi apparatus was visualized with anti-GRASP65 (Abcam) and hippocampal pyramidal cells were identified with anti-Ctip2 (Abcam). Goat anti-mouse-HRP, donkey anti-rabbit-HRP, biotin-conjugated donkey anti-mouse-HRP, FITC-conjugated Streptavidin, were purchased from Jackson Immunoresearch, and Alexa fluor 488-conjugated donkey anti-rabbit and Alexa fluor 568-conjugated donkey anti-rat were from Invitrogen.

The Stk25 shRNA and triple-point-mutant shRNA control have been described previously [34], and were expressed from the U6 promoter in the pLL3.7 lentiviral vector (empty vector), which also expresses GFP from a CMV promoter/enhancer [47]. High titer virus was produced as described previously [14] and used to infect 90–100 percent of the neurons the Tau phosphorylation experiments.

The vectors pCAGGS-Stk25GFP [34] and human Tau 2N4R [48] were used for the HeLa cell transfection experiment. The GFP fusion protein of Stk25 was chosen to facilitate the rapid assessment of transfection efficiencies. We have also used an HA-Stk25 fusion in similar experiments to that shown in Figure 4B. We have confirmed the kinase activity of the HA-tagged fusion of Stk25 by in vitro kinase assay (data not shown).

### Neuronal characterization

Neurons were cultured essentially as described [34]. Neuronal polarity was assessed in hippocampal neurons that were infected with low titer GFP expressing virus on the day of culturing and then replated two days later on a poly-L-lysine coated coverslips, which were placed over a monolayer of astrocytes. Neurons were fixed in 4% paraformaldehyde for 10 min at room temperature, after 6 days of culture. Axons were defined as SMI-312 positive processes that were greater than 250 µm in length.

The Golgi apparatus was visualized with anti-GRASP65 antibody in sections of P0 mouse brain perfused with 4% paraformaldehyde [34]. Golgi extension was measured from the edge of the nucleus of Ctip2 positive pyramidal neurons to the tip of the furthest extent of the Golgi.

### Western Blotting and Immunoprecipitation

Postnatal day 40 (P40) hippocampi were collected for Western blot analysis. For Tau analysis of hippocampal samples in Fig. 2, the tissue was sonicated in homogenization buffer (0.1 M MES [pH6.8], 0.5 mM MgSO4, 1 mM EGTA, 2 mM dithiothreitol, 0.75 NaCl, 2 mM PMSF, 20 mM sodium fluoride, 0.5 mM sodium orthovanadate, 1 mM benzamidine, 25 mM β-glycerophosphate, 10 mM p-nitrophenylphosphate, 10 mg/mL aprotinin, 10 mg/mL leupeptin, and 1 mM okadaic acid) and clarified by centrifugation at 14,000 g at 4°C. Samples were heat denatured in a boiling water bath for 5 min and then chilled for 5 min. For Tau analysis of the whole brain samples in Fig. 4 the tissue was homogenized in RIPA buffer (Sigma) supplemented with a cocktail of protease and phosphatase inhibitors (Roche). Samples were then centrifuged at 14,000 g for 5 min to remove precipitated protein. Equal amounts of protein, as determined by Bradford assay, were diluted with an equal volume of 2× sample buffer (4% sodium dodecyl sulfate, 40% glycerol, 0.2 M Tris-HCl, [pH 6.8], 5.6 M 2-mercaptoethanol, 5 mM EDTA, 0.02% bromophenol blue), heat denatured at 100°C for 5 min, resolved by polyacrylamide gel electrophoresis and Western blotted.

For anti-Dab1 Western blots, brain-samples were sonicated in RIPA lysis buffer (50 mM Tris–HCl, 50 mM NaCl, 0.25% sodium deoxycholate, 1 mM EGTA, 1% Igepal, 20 µg/mL PMSF, protease inhibitor cocktail [Roche]) for 10 min on ice. Samples were diluted in sample buffer, resolved on 4–12% Bis-Tris-gradient gels (Invitrogen) and electrotransferred to PVDF membranes (Millipore, Bedford, MA). Membranes were blocked in 5% milk dissolved in Tris-buffered saline with 0.1% Tween 20 (TTBS) and incubated with indicated primary and secondary antibodies. Signals were detected with X-ray film from membranes soaked in enhanced chemiluminescence reagent (ECL, Invitrogen).

To examine phopho Tau levels in cultured neurons, cell lysates were prepared with RIPA buffer (20 mM Tris-HCl [pH 7.4], 0.15 M NaCl, 1% Nonidet P-40, 2 mM EDTA, 1% sodium deoxycholate, 0.1% SDS, 5 mM 2-mercaptoethanol, 50 mM sodium fluoride, phosphatase inhibitor cocktail 1 [Sigma], 1 mM phenylarsine oxide [Sigma], and protease inhibitors [complete mini, EDTA free; Roche]) from 1×10^6^ cortical neurons grown in 1 well of a 12 well dish. Cells were infected with the indicated viruses 3 days before cell lyses as described previously [14].

### HeLa cell transfection

One day after HeLa cells were plated (1×10^6^ cells per chamber of a 6 well dish), cells were transfected with 2 µg of each indicated DNA using FuGene HD (Roche Applied Science) according to the manufacturer's directions. Two days after transfection the cells were lysed in homogenization buffer (see Western blotting above), and cell lysates were prepared as described for the hippocampus above.

### Immunohistochemistry

To image Tau phosphorylation in the hippocampus, isolated brains were cryoprotected in 40% sucrose in PBS, and frozen sections were prepared with a cryostat (30 µm thick). Sections were incubated with the AT8 antibody, followed by a biotinylated secondary and then Streptavidin-FITC. Images were collected with a DeltaVision Deconvolution microscope (Applied Precision).

### Microarray

Profiling of >45,000 mRNA transcripts was performed using MouseWG-6 Expression BeadChips (Illumina Inc.) as previously described [49]. Briefly, total RNA from individual samples were converted to cRNA using the Illumina® TotalPrep RNA Amplification Kit (Ambion Inc., Austin, TX) and then 1500 ng was hybridized to the arrays according to manufacturer's protocols. Raw intensity values for each probe were transformed using the rank invariant normalization method [50] within the Beadstudio analysis software. The results are available online (GEO, GSE22044).

### Real-Time PCR

Total RNA was prepared from hippocampi of P19 mice using an RNeasy RNA extraction kit (QIAGEN, Chatsworth, CA). Single-stranded cDNA was prepared from total RNAs using an Oligo (dT)12–18 primer and Superscript III reverse transcriptase (Invitrogen). RT-PCR analysis was done with the Power SYBR green PCR Master Mix kit and an ABI PRISM7900HI real-time PCR system (Applied Biosystems), using the following primer sets: Stk25, GACCGATATAAGCGCTGGAA and TGTCTGAGATGCCAGGACAG; for β-actin, GACGGCCAGGTCATCACTAT and ACATCTGCTGGAAGGTGGAC. Stk25 expression data were normalized to β-actin levels and averaged across three separate experiments done in triplicate with three independently generated RNA samples.
